# Research capacity and training needs for non-communicable diseases in the public health arena in Turkey

**DOI:** 10.1186/1472-6963-14-373

**Published:** 2014-09-05

**Authors:** Bulent Kilic, Peter Phillimore, Duygu Islek, Dilek Oztoprak, Eren Korkmaz, Niveen Abu-Rmeileh, Shahaduz Zaman, Belgin Unal

**Affiliations:** Department of Public Health, Dokuz Eylul University Faculty of Medicine, 35340 Izmir, Turkey; Department of Sociology, Newcastle University, Newcastle Upon Tyne, UK; Department of Public Health, Dokuz Eylul University Institute of Health Sciences, 35340 Izmir, Turkey; Institute of Community and Public Health, Birzeit University, Occupied Palestinian Territory, oPt, Ramallah, Palestine; Institute of Health and Society, Newcastle University, Newcastle Upon Tyne, UK

**Keywords:** Training needs assessment, Research capacity, Mapping, Non-communicable diseases, Public health

## Abstract

**Background:**

The aim of this study is to define the research capacity and training needs for professionals working on non-communicable diseases (NCDs) in the public health arena in Turkey.

**Methods:**

This study was part of a comparative cross-national research capacity-building project taking place across Turkey and the Mediterranean Middle East (RESCAP-Med, funded by the EU). Identification of research capacity and training needs took place in three stages. The first stage involved mapping health institutions engaged in NCD research, based on a comprehensive literature review. The second stage entailed in-depth interviews with key informants (KIs) with an overview of research capacity in public health and the training needs of their staff. The third stage required interviewing junior researchers, identified by KIs in stage two, to evaluate their perceptions of their own training needs. The approach we have taken was based upon a method devised by Hennessy&Hicks. In total, 55 junior researchers identified by 10 KIs were invited to participate, of whom 46 researchers agreed to take part (84%). The specific disciplines in public health identified in advance by RESCAP-MED for training were: advanced epidemiology, health economics, environmental health, medical sociology-anthropology, and health policy.

**Results:**

The initial literature review showed considerable research on NCDs, but concentrated in a few areas of NCD research. The main problems listed by KIs were inadequate opportunities for specialization due to heavy teaching workloads, the lack of incentives to pursue research, a lack of financial resources even when interest existed, and insufficient institutional mechanisms for dialogue between policy makers and researchers over national research priorities. Among junior researchers, there was widespread competence in basic epidemiological skills, but an awareness of gaps in knowledge of more advanced epidemiological skills, and the opportunities to acquire these skills were lacking. Self-assessed competencies in each of the four other disciplines considered revealed greater training needs, especially regarding familiarity with the qualitative research skills for medical anthropology/sociology.

**Conclusions:**

In Turkey there are considerable strengths to build upon. But a combination of institutional disincentives for research, and the lack of opportunities for the rising generation of researchers to acquire advanced training skills.

## Background

A strong research capacity is very important to finding the causes of diseases and sustaining a healthy life for all. Research capacity plays a central role in any health system and allows for building new evidence for policy-making. The United Nations Development Program (UNDP) definition of research capacity strengthening focuses on following key elements: strengthening the abilities of individuals, institutions and countries to perform research functions, defining national problems and priorities, solving national problems, utilizing the results of research in policy-making and programme delivery [1]. A summary of major issues observed in developing countries in this area reveal six major gaps and deficiencies: Low priority for research, lack of prioritization of research problems, lack of research findings application in policy processes, lack of applied knowledge, non-optimal use of human resources and issues with monitoring and evaluation of research results [2].

Academia, policymakers, and NGOs (non-governmental organizations) are crucial partners in this process. The World Health Organization (WHO) refers to the triangular relationship among these agencies as a *“global stakeholder alliance”* necessary for a public health workforce [3]. Research capacity building in public health in Turkey is important because of the recent demographic transition resulting in an aging population with increasingly serious health problems such as non-communicable diseases (NCDs). Despite the need, however, there is no previous research in the public health arena in Turkey, examining training needs in relation to NCDs for public health researchers and policy makers. The aim of this study is, first, to describe a method for defining research capacity and the training needs for junior researchers on NCDs in public health, and second, to present results from a larger study on Turkey.

In an analysis of PubMed articles on health workforce training published between 1970 and 2004, over 90% of articles focused on educational measurement, teaching methods or curriculum issues [3]. According to the WHO, research is urgently needed on other aspects of health workforce training, including skills, training needs and prevalence of fellowships. Making better use of health services research in developing public policy requires that both health services researchers and public policymakers should have realistic goals and priorities [4]. Around the world, policymakers have identified human resources as the area of the public health systems most in need of investment, and the first priority for health system strengthening [3]. In addition to human resources, financing and NGOs are also important themes, and there are significant gaps in existing training programmes [5].

Though Turkey has been increasingly emphasizing the importance of research, it is evident in international comparisons that it can do better. The average expenditure for research is 0.85% of GDP for years 2005-2009 [6] and 0.86% in 2011 in Turkey [7]. The percentage of expenditures for research of GDP is 4.27 in Israel, 3.96 in Finland, 2.79 in USA, 1.87 in UK, 1.10 in Tunisia, 1.08 in Brazil, 0.79 in Iran, 0.58 in Greece, 0.52 in Argentina, and 0.42 in Jordan [6]. Expenditure for research in Turkey is lower than that of similar countries like Tunisia, Israel and Brazil but is higher than Greece, Iran, Jordan, and Argentina. However, research capacity in Turkey remains insufficient compared to many Western countries. For example, between 2005-2009, while the number of full-time researchers was 804 per million people in Turkey, it was 7,647 in Finland, 4,673 in USA, 3,947 in UK, 1,863 in Tunisia, 1,849 in Greece, 1,046 in Argentina, 751 in Iran, and 696 in Brazil [6]. According to these figures, Turkey is ranked middle to low by comparison in terms of research capacity.

Turkey did not have a formal national health research framework until recently. In 2012, the Turkish Ministry of Health (MoH) was reorganized and the General Directorate of Health Research was newly established [8]. This was a very late step for Turkey, and it is clear that the Turkish health research system is not well developed when compared to Western countries. For example, the English health research system was established in 1960s, and since the 1970’s has undergone four main phases of reform [9]. In addition, the number of researchers who work in public health area in the Turkish MoH are quite small [10] and most importantly research priority areas have not yet been defined at national level [11]. This situation is similar to other developing countries. Research capacity strengthening, health research framework and priority setting in the developing countries of the Eastern Mediterranean Region and particularly low and middle income countries are not well developed and often weak [2, 12, 13].

On the other hand, the strategies and priorities of Turkey are changing very rapidly. According to the strategic plan of the Turkish MoH, NCDs are now a priority problem for Turkey for the years of 2010-2014 [14, 15]. Ischemic heart disease is the number one cause of death, accounting for 22% of all deaths in Turkey [16]. Among the twenty major diseases which cause the highest Disability Adjusted Life Years (DALY) at the national level, ischemic heart disease occupies second place (8%) overall [17]. According to Turkey’s latest data on diabetes, the prevalence of Diabetes Mellitus (DM) was 16.5% (undiagnosed 7.5%) in 2010, indicating 6.5 million adults with DM in Turkey according to the latest estimates [18]. Mediterranean studies of cardiovascular disease and hyperglycemia project (Med CHAMPS), which included Turkey, suggested that research capacity on NCDs should be strengthened in order to face the rapidly increasing incidences of NCDs in Turkey [19]. Building and strengthening research capacity requires a situational analysis and training programs. Training needs should be identified before the development of training programs. In order to establish priorities, to create stakeholder commitment, to analyze value for money and in order to monitor and evaluate outcomes, systematic training needs assessment should be conducted [20].

There are also a considerable number of Research Institutes of Health under the universities and NGOs (associations, foundations and international organizations) with a concern for NCDs and public health. In addition, at the first level, Provincial Directory of Public Health; at secondary level, State Hospitals with basic clinic branches; and at tertiary level, Training and Research Hospitals and University Hospitals provide training and research in addition to curative services. However the training needs of researchers and priorities in research areas in relation to NCDs in Turkey are not known. The originality of this study, which was part of a comparative international study, is to focus on health inequalities and social determinants of NCDs and to define the training needs in this area using a mixed methods approach (integrating qualitative and quantitative techniques).

## Methods

**Figure 1:**
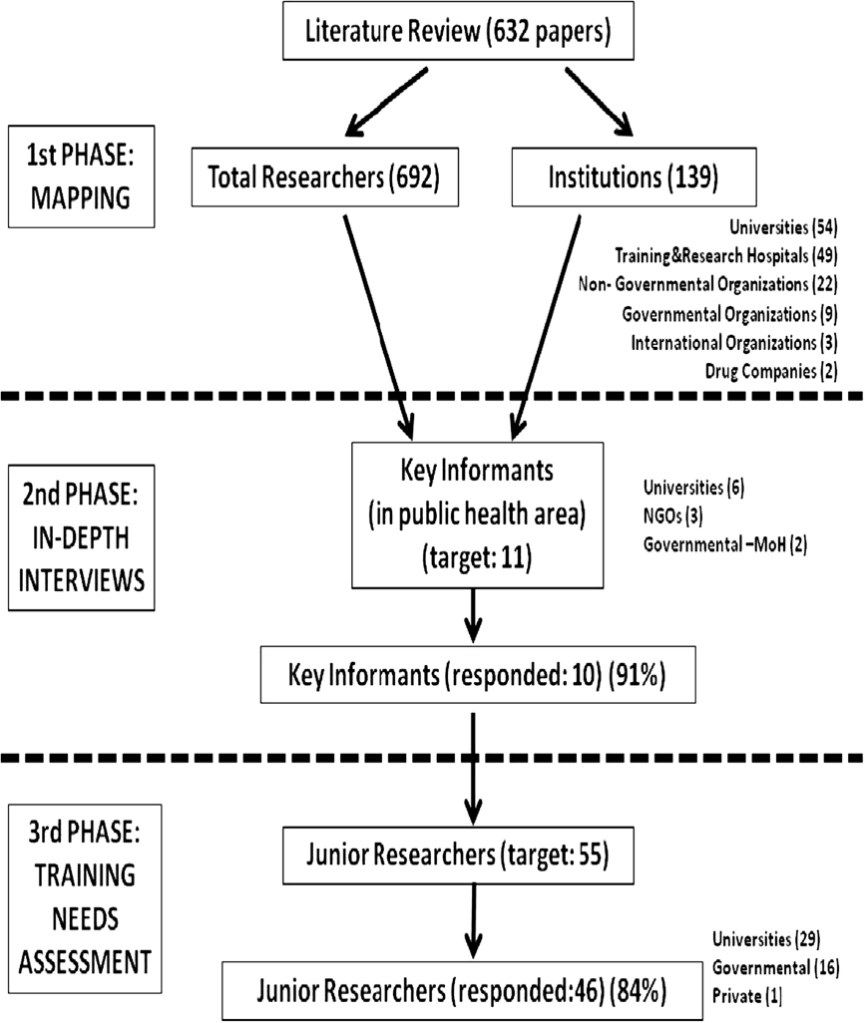
**Research design.**

This study is a part of the European Commission FP7 funded project RESCAP-Med that aims to build public health research capacity in social determinants of NCDs in Turkey, Palestine, Lebanon, Syria, Tunisia and Jordan [21]. This study refers specifically to the training needs assessment component, and not RESCAP-Med as a whole. Our work, which has three phases, is part of a comparative cross-national study which is designed to build on each other systematically. This study was conducted between April-October 2012 in Turkey, employing a mixed methods approach. The research design and sequence is described in Figure 1.

Multi-phase and mixed method approach in this study make this research an innovative one by combining the “training needs assessment” method adapted from Hennessy-Hicks [22] and “mapping” method adapted from WHO (WHO 2008). The five disciplines in research and training area identified at the beginning of project were: epidemiology, health economics, medical sociology-anthropology, health policy, and health environment. These disciplines were pre-identified in the proposal to the European Commission for special consideration and that decision was based on the findings of the MedCHAMPS Project [19]. Project countries and the central team discussed the method for the training needs assessment in a workshop which took place in Jordan in May 2012. Coordinators from different countries negotiated and fine-tuned the initial design, and, since this is a comparative study, teams discussed what was feasible in each national setting, especially given the short timescale needed to pave the way for RESCAP-Med’s events and trainings (because this stage was a baseline for the rest of the project). The overall coordinating role fell on the Palestinian team and the RESCAP-MED project coordinator was chosen from Birzeit University, Palestine.

The first phase of the study included mapping institutions involved in health related research and largely adapted the methodology of “National Health Research System in the Eastern Mediterranean Region” study which was conducted by WHO in 12 Eastern Mediterranean Countries in 2008 [23]. A short version of the coding schema of the above mentioned study was used with slight modifications to understand the national health research system in Turkey and to provide a database of research in the field of NCDs. Using this form, institutes that conduct research and/or commission research were investigated. These institutions are categorized as government institutions, training and research hospitals, universities, NGO’s, for profit private institutions and international institutions which sponsor research in Turkey. In addition to these, institutes which are initiators of research in Turkey, the national surveys conducted throughout the country, the institutes that are responsible for organizing these national studies and the strategies for this goal were also coded in the data form.

At the stage of compiling the literature database, our team first searched papers authored by Turkish researchers in the field of NCDs on PubMed for SCIE and SSCI journals, using key words ‘coronary artery diseases’, ‘cardiovascular diseases’, ‘cerebrovascular diseases’, ‘diabetes mellitus’, ‘hypertension’, ‘metabolic syndrome’, ‘stroke’, ‘dislipidemia’, ‘obesity’, ‘nutrition’, ‘diet’, ‘physical activity’, ‘exercise’, ‘health inequalities’, ‘social determinants of health’ and ‘Turkey’ between the years 2000- June 2012. Resulting search was first coded by the name of the institutes where the research was conducted. In the second stage, number of studies by each institution was entered into the data form. At this stage, whether these institutions had conducted the studies themselves or whether they had only commissioned research from others was taken into consideration. Researchers who had authored the highest number of research publications were listed. Finally, from this list, we identified seven researchers who conducted research in the field of ‘public health’ and particularly in the field of ‘the social determinants of health’ as our Key Informants (KIs) for the second phase of the study. Additionally we identified two editors of scientific journals and two senior representatives from the department of NCDs at the Ministry of Health (the primary institution with responsibility for managing NCDs), and the General Directorate of Health Research (the primary institution with responsibility for national health research in Turkey) as KIs as well.

In the second, qualitative, phase we were able to interview 10 of the 11 KIs who were identified during the mapping phase (literature review) in six Turkish cities: Istanbul, Ankara, Izmir, Bursa, Eskisehir and Manisa. Aside from the two senior policymaker KIs from MoH the rest of KIs are academics from departments of public health and biostatistics (five people) and representatives from NGOs (two senior editors of Turkish scientific journals and one president of the national public health congress) who play a significant role in the research area on NCDs and public health. In these interviews, we focused on questions regarding the strategic aims of the institutions and future research plans along with questions about the Turkish health research system and training needs of young researchers in NCDs in the public health arena. All interviews were carried out at the KIs’ working place, and lasted around one hour. All interviews were taped with permission. All tape recordings were transcribed. They were then coded and analyzed for recurring themes.

The third phase was quantitative and the sample was gathered through snowball and theoretical sampling techniques. KIs from the second phase were asked to suggest suitable respondents for this phase. A Training Needs Assessment (TNA) questionnaire was then sent by email to 55 researchers (from 15 different institutes and 10 cities). Forty six researchers responded (84%). TNA is an approach devised by Hennessy-Hicks [22] and it was revised and adapted by the central and coordinator research teams [21] to make it appropriate for NCDs, including their social determinants, and the five selected research disciplines. The Hennessy-Hicks instrument is unique in that it is tailored for use specifically with health care teams but can easily be adapted to meet particular objectives in this case for research training needs. Respondents score each item in the instrument for importance and performance. The “Importance Rating” seeks to address how important the junior researcher perceives the given activity is for research. The rating measurement to be used in the “Importance Rating” is 1-7, with 1 indicating that the task is not at all important and 7 that it is very important to research. The “Current Performance Rating” is concerned with the junior researcher’s own mastery of the specific activity. The rating measurement to be used in the “Current Performance Rating” is 1-7, with 1 indicating very limited capability and 7 indicating very strong capability. Importance compared with performance provides an assessment of where the greatest training needs lie. The biggest gap indicates the greatest training need [22]. Descriptive statistics such as means and standard deviations were calculated for every item. Training needs were defined as the difference between the importance and performance scores. Training needs and competence are also summarized as the percentages of participants who answered “yes” for additional “yes/no” questions for every sub competency.

Ethical statement has been approved for this research by Izmir Clinical Researches Ethical Committee (no:B.30.2.EGE.0.20.00.05.OY/1502-1218).

## Results

### Mapping

According to the literature search conducted in PubMed, there are 632 articles authored in Turkey and published in journals between January 2000 and June 2012 in the field of NCDs. After coding the articles in relation to their main subject, there were 154 articles on the subject of hypertension, 129 articles on the subject of diabetes mellitus, 122 articles on the subject of obesity, and 113 articles on the subject of coronary artery diseases. The least prevalent topics were: 28 articles on the subject of cerebrovascular diseases, 23 articles on the subject of metabolic syndrome and 13 articles on the subject of physical activity. In terms of institutions, training and research hospitals and NGOs appear to make the largest contribution to the different areas (compared with universities), with their main themes being coronary artery disease (27.4%) and metabolic syndrome (21.5%) in hospitals; and dyslipidemia (16%) for NGOs. All of the physical activity papers had been published by universities (Table 1).

**Table 1:** **Distribution of papers according to subject and institutions in Turkey (2000 January-2012 June)**

Paper subject	Universities		Hospitals		NGOs		Total	
	n	%	n	%	N	%	n	%
Hypertension	128	83.1	20	13.0	6	3.9	154	24.4
Diabetes Mellitus	107	82.9	17	13.2	5	3.9	129	20.4
Obesity	104	85.2	10	8.2	8	6.6	122	19.3
Coronary Artery Disease	70	62.0	31	27.4	12	10.6	113	17.9
Dyslipidemia	37	74.0	5	10.0	8	16.0	50	7.9
Cerebro Vascular Disease	22	78.6	5	17.9	1	3.5	28	4.4
Metabolic Syndrome	16	70.0	5	21.5	2	8.5	23	3.6
Physical Activity	13	100.0	-	-	-	-	13	2.1
**Total**	**497**	**78.7**	**93**	**14.7**	**42**	**6.6**	**632**	**100**

However, research on NCDs is usually focused on clinical studies which evaluate the effectiveness of medical interventions, and overlook the social determinants, prevention, health promotion and public health aspects of disease. Moreover, existing publications are usually cross-sectional and consist of quantitative epidemiological surveys. Research on NCDs which focus on social determinants and use qualitative techniques in the public health arena are rare.

Overall, 497 studies (79%) were conducted by 54 different universities. It was also found that 49 training and research hospitals had conducted 93 studies (19%) in the NCDs field. NGOs also perform an important role in conducting research on the NCD field. Twenty-two NGOs were found to have conducted 42 studies. When 9 government institutions were examined, the most important institutions were the MoH, the Scientific and Technological Research Council of Turkey (TUBITAK) and the Turkish Statistical Institution (TUIK) which are partner institutions of the other institutes (usually universities).

The key national multi-sponsored projects were TEKHARF [24], TURDEP [18, 25], TOHTA [26], Burden of Disease [17], Turkey Demographic and Health Survey [27, 28] and BAK [29, 30]. Institutions that support health research respectively are Scientific and Technological Research Council of Turkey (TUBITAK), the MoH, and the Higher Education Council (YOK). TUBITAK provides scholarships, grants and awards. Studies such as “Turkey’s Burden of Disease”, “National Chronic Diseases and Risk Factors” as well as other research were also carried out with the support of the MoH. The Higher Education Council in Turkey provides a budget for universities and research. The number of researchers who conduct research in the field of NCDs was also investigated. Six hundred and ninety-two researchers were found to have conducted research in NCDs. After this search, when we added two new search terms: “health inequalities” and “social determinants of health”, we only found 31 articles in our database of 632 papers (about 5%). This indicates a lack of research focus regarding health inequalities or social determinants of disease. We conclude that the public health field in Turkey does not find the field of health inequalities and social determinants of NCDs as important as burden of disease. The role of social sciences in public health is still in its infancy in Turkey, as in many other countries.

Among the 15 training courses held in the last few years, only three were about NCDs: Training on diabetes mellitus for MoH workers, MEDCHAMPS project workshops, a TUBITAK summer course on research design on cardiology, epidemiology courses (presented 10 times by the MoH for MoH workers), biostatistics (university based), and qualitative research design (university based). The MoH, TUBITAK, EU, and universities financially sponsored these training workshops. There was only one international training project on NCDs: the MEDCHAMPS project in Turkey.

### Judgements of Key Informants (KI)

Interviews with KIs revealed that research capacity building in relation to NCDs in public health area is important for Turkey: Eight of the 10 KIs think this topic is very important while the other two identified this as moderately important.

Analysis of the interviews identified institutional and individual factors that contribute to the current state of research in Turkey in this area. Institutional and structural factors appear to be more prominent in their judgments. Institutional factors mentioned included required research component in promotional consideration, restructuring of the MoH, the new performance based payment system, teaching heavy workloads, lack of financial support, lack of specialization and prioritization, lack of coordination among stakeholders as well as a lack of support network among researchers which highlights the problems with dissemination of research and application at the administrative levels. Individual factors mentioned included a lack of motivation and curiosity on the part of researcher as well as inadequate language skills, specifically English.

First, we will discuss individual factors, and then devote the majority of our analysis to institutional barriers. Achieving a research capacity comparable to international standards require that researchers can in fact engage with the international community of researchers and are familiar with their methodologies and literature in general. To that end, one KI proposed that training abroad would be very important by providing a chance to observe academic life in another country. This suggests a fluency in English at the least is required, especially when the training language is other than Turkish. All of the KIs in our study support sending staff abroad for one to two months. Four KIs mentioned that training abroad would contribute greatly to the professional development of a young researcher, and it is an academic requirement at some institutions. For example one KI mentioned that

*“…Via a short presentation, we wanted our residents to share information they had learned from a course which had been conducted in English. There was a small problem. They said that they did not understand some parts of the course and therefore could not report back very well. ….Generally, most junior researchers are not fluent enough to follow a course in English. …The language problem is an important barrier.”Academician*

Most KIs mentioned that training of junior researchers towards research capacity building in NCDs will contribute to the overall mission of their institutes by improving staff motivation, increasing knowledge base and generating new ideas. However, while half of the KIs thought that junior researchers desire such training, other half disagreed that there was enough demand from junior researchers. There seems to be a disconnect between the KIs and junior researchers regarding demand for training since 70% of the junior researchers surveyed in the next phase stated a desire for training. The KIs who believed in a lack of demand argued that junior researchers have low motivation and they lack curiosity for research. This suggests that the reason for low research production and low researcher numbers in Turkey stems from individual factors, indicating a belief in internal motivation as necessary. Only one KI mentioned that the reason for lack of interest is a reflection of the expectations of their institution.

*“There is no interest in research, they want someone else to conduct research and they only want to read it. If they are interested in research, the reason is this: if they do not have publications, they will not be promoted as a professor or associate professor. A thesis for specialization is compulsory, because if they do not have a thesis, they will not be a specialist in medicine. Indeed, there is no curiosity.”Editor of a SCIE scientific journal*

What is interesting in this quote is that while the KI points to a lack of curiosity for research, which is an individual factor, he also suggests that institutional requirements for research for promotional reasons are indeed a supporting factor for research. However such a requirement may not be enough to spur research activity as KIs from medical schools brought up heavy teaching workloads as a barrier.

*“The main problem for public health researchers is* the heavy load of *undergraduate education in medical schools. So, there is no time for research, really… We have a very heavy teaching commitment. It is too hard for a researcher to take a full-time role in a project, even if it is short-term.”Academician*

KIs also mentioned that heavy undergraduate and postgraduate educational tasks are, in addition to taking too much time away from research activity, also the culprit for inadequate specialization and hence low productivity.

*“Academics who are focused on only one field bring the science more up to date and are more productive…. I mean, if there could be researchers working only on one area like heart disease, strokes, or diabetes and if there were no other expectations from them, we would have an opportunity to reach the level of Western countries … We have a problem of not focusing or concentrating enough on one subject…”Academician*

It seems likely that even the individualized factors mentioned above (lack of language skills and motivation for research) cannot be explained indirectly through structural factors. Indeed, these factors are closely related. For example, opportunity to train abroad where language proficiency achieved and where the researcher becomes familiar with other research contexts and processes, and has a chance to establish a network, could in fact initiate and perhaps sustain a momentum for research activity upon return through increased self esteem and motivation. Structural factors mentioned by KIs in this study are multi faceted and differ based on the institutional setting (governmental vs. non-governmental vs. medical schools/hospitals).

Larger issue regarding training in the current context is the reorganization of the national health research system, which is incomplete and created a transitional stage. Historically research at MoH which was rare was decentralized and uncoordinated. Governmental KIs (from the MoH) acknowledged this issue but stated that they were hopeful for future.

*“…….the General Directorate of Health Research has been newly established and has a history of only five or six months. ….Historically, our staff [of MoH] rarely conduct research, after all, it wasn’t planned this way…but we are hopeful for the future.”Senior Policymaker*

Lack of coordination among institutions and specifically between the academics, NGOs and the government appear to be a key problem identified by the KIs.

*“..There is no relationship between academia and the MoH. The MoH does not state a need for research in any particular field or a need for evaluation of any particular policy. The MoH does not want to have this kind of connection with universities. But people we trained go on to work in the MoH later. In fact, the MoH can give them this kind of responsibility. It can require them to conduct research or evaluate a policy. If there was such a connection during training, the junior researcher could do this more effectively when he goes there. ..There is a lack of collaboration, such as working together on implementation, or working towards solutions for real problems…”Academician*

Academic KIs’ concern for lack of collaboration and coordination is not shared by the governmental KIs. For example one policymaker KI mentioned that they found their arrangement sufficient for their procedures.

*‘…….Conducting research is the duty of General Directorate of Health Research. We think that this makes our task easier. For instance, when we wonder about the effectiveness of our new interventions and want to evaluate this, the General Directorate of Health Research is the first place that we contact…’Senior Policymaker*

Another issue brought up is the lack of applied knowledge in the field by the MoH. If trainees are not in a position to implement what they learned at their workplace as part of their daily workload, then the training itself becomes a moot point and further diminishes motivation.

*‘“… So the biggest problem is this: there is no way of keeping people at the high level of motivation that they have reached after these courses. So she came, learned and was very excited, but the day after the training she goes to her department and does nothing [related to the training she received]. Her position will not be adequate, and she will have no opportunities or access to a network. I mean, there is actually no national health research system to speak of…..”Academician*

This lack of network also points to an issue with the dissemination of research results, which was identified as inadequate in the Turkish context by the KIs in this study. Turnover is another problem, aside from the inability to apply what is learned as part of work responsibilities. One KI mentioned that:

*“Last year, we trained 250 people, who were staff of the MoH, in the field of epidemiology and expected that they would work in specific departments which were related to epidemiology and make a contribution as epidemiologists. But one third of them did not stay in those departments.”Academician*

Another significant barrier is the financial resources available for research. KIs specifically mentioned the new performance based payment system (in 2011 for the universities) and its negative effects on research activities by discouraging health workers to spend time on research, instead focusing on clinical work to increase their take home pay.

*“… and in the end, there came the system of payments based on performance and whatever happened, people stopped bothering to undertake research. Every person is willing to see a patient rather than conducting research…”Editor of a SCIE scientific journal*

Aside from governmental resources, there is the issue of diminishing financial support from the for-profit sector, such as pharmaceutical companies.

*“…The first is really a financial problem. … Formerly we have been receiving great support from pharmaceutical companies. Now, in order to increase their profit share they have become almost unable to support us. Furthermore, even when there is such a research project, namely if it is not prescribed [i.e. increase drug sales], they do not give the researcher anything…”NGO Representative*

Bureaucracy of getting research clearance is another issue. When researchers try to involve governmental or private institutions in their research, in addition to the lack of grants available, they may not receive access to data or permission to pursue it.

*“Recently, we wanted to conduct a study about family medicine at the level of primary care, but we could not get permission from the MoH. Permission problems can occur elsewhere, too”.NGO Representative*

Lastly, lack of prioritization of research topics is especially problematic according to the interviews, and lack of coordination and collaboration is evident between academia and the government.

*“…Nobody knows how to access the information gathered by the MoH. There is a disconnect. We don’t know what the MoH wants, or areas of demand. We don’t even know if their data can meet their needs….”Academician*

In summing up, our KIs mostly identified infrastructural and institutional issues as barriers against research capacity building in NCDs in public health in Turkey, which may also indirectly contribute to the few individual factors mentioned earlier.

In the next section, we present our findings from the quantitative surveys completed by junior researchers.

### Training needs of junior researchers

Fifty-five junior researchers from 15 different institutes were invited to complete the Training Needs Assessment (TNA) questionnaire by e-mail, and 46 researchers responded (84% response rate). Junior researchers were defined as young and mid-level researchers who had one of the following: a medical degree and residency at the university, or a bachelor’s degree and experience in health research, or a master’s degree in health (or a related discipline) or a PhD degree and a maximum of seven years of experience in the field. Some of them were young academics or specialists in medicine. Their specialization is mostly in public health, with one cardiologist and one obstetrician-gynecologist. Sixty-one percent of junior researchers in our sample are female medical doctors working at a university, and half of them have postgraduate education. The mean age of the junior researchers is 33 and their average work experience is nearly four years at their most recent institution (Table 2).

**Table 2:** **Socio demographic findings of junior researchers**

(n = 46)		N	%
Gender	Female	28	61.0
	Male	18	39.0
**Highest level of education completed**	**University (Medical Faculty)**	**22**	**47.8**
	**PhD (Public Health)**	**10**	**21.7**
	**Specialization in Medicine**	**9**	**19.6**
	**MPH (Master of Public Health)**	**5**	**10.9**
Institutional affiliation	University	29	63.0
	Ministry of Health	15	32.6
	Ministry of Labor and Social Security	1	2.2
	Private (Occupational Physician)	1	2.2
**Job title**	**MD (Resident/Research Assistant)**	**22**	**47.8**
	**MD (Specialist)**	**8**	**17.4**
	**Academician**	**8**	**17.4**
	**Nurse**	**2**	**4.3**
	**Anthropologist**	**2**	**4.3**
	**Dietician**	**1**	**2.2**
	**Psychologist**	**1**	**2.2**
	**Engineer**	**1**	**2.2**
	**Health Officer**	**1**	**2.2**
		Mean	SD
Age		32.9	5.9
Working years		3.7	4.0

When we evaluated general skills and qualifications, computer skills of junior researchers (Microsoft Office Power Point, Word, e-mail, internet etc.) were usually above average except in Excel, and statistical software as self reported. When English competence was considered, junior researchers had particular problems in writing and speaking in English. The language skills among 15% of junior researchers in the sample were below average in written English, and spoken English skills were below average among 20% of junior researchers. In addition to language problems, 15% and 26% of junior researchers had below average skills in Excel and statistical software (such as SPSS) respectively.

Tables 3 and 4 clarify the distinction between generic skills and discipline-specific skills. Table 3 deals with tasks pertaining specifically to research and scientific writing skills, which is necessary for all research endeavors. Average performance scores for research design and implementation were usually over four points (over a seven point scale), except for qualitative study design and use of qualitative research tools. This means that junior researchers do not need research design training except in qualitative research. Average performance scores were similar for analysis and writing competencies, except for qualitative report writing, qualitative data analysis and policy paper and academic journal writing. The “gap” relates the difference between the two columns (importance and performance scores). The biggest gap was in qualitative report writing and qualitative data analysis (2.9 points). The last column in Table 3 is “training need,” which is the percentage of junior researchers who answered “yes” to the question “do you need training in….?”. The highest training need - approximately 90% of junior researchers – was identified as training in how to write a policy report (Table 3).

**Table 3:** **Junior researchers’ perception of barriers in research knowledge**

***(n = 46)***	***Importance score mean ± SD***	***Performance score mean ± SD***	Gap* (difference) mean ± SD	***Training need %***
***Research design & implementation***	***6.2 ± 0.9***	***4.5 ± 1.4***	***1.7 ± 1.4***	***66***
Study Design: Qualitative	5.9 ± 1.4	3.4 ± 1.8	2.6 ± 2.0	80
Qualitative Research tools	5.8 ± 1.3	3.7 ± 1.7	2.1 ± 1.8	77
Writing research proposals	6.4 ± 1.0	4.5 ± 1.6	1.9 ± 1.5	66
Data management	6.5 ± 0.8	4.8 ± 1.7	1.8 ± 1.8	61
Study Design: Quantitative	6.2 ± 1.2	4.5 ± 1.6	1.7 ± 1.6	70
Routine/secondary data use	6.2 ± 1.2	4.5 ± 1.7	1.7 ± 1.8	68
Ethical guidelines & oversight	6.3 ± 1.2	4.9 ± 1.5	1.4 ± 1.9	64
Questionnaire development	6.1 ± 1.3	4.7 ± 1.7	1.3 ± 1.6	54
Conducting literature reviews	6.5 ± 0.8	5.2 ± 1.4	1.3 ± 1.6	61
***Analysis & writing***	***6.3 ± 0.9***	***3.8 ± 1.4***	***2.5 ± 1.6***	***80***
Writing qualitative reports	6.1 ± 1.2	3.1 ± 1.8	2.9 ± 2.0	82
Qualitative data analysis	6.1 ± 1.2	3.2 ± 1.8	2.9 ± 1.9	84
Writing policy papers	5.9 ± 1.4	3.3 ± 1.7	2.7 ± 2.0	89
Writing academic journal articles	6.5 ± 0.9	3.9 ± 1.9	2.6 ± 2.0	79
Writing quantitative reports	6.3 ± 1.0	4.1 ± 1.9	2.2 ± 1.9	75
Statistical analysis	6.5 ± 0.8	4.4 ± 1.7	2.1 ± 1.6	77
Conference presentation skills	6.4 ± 0.8	4.4 ± 1.7	1.9 ± 1.8	73

* The gap refers to the difference between the importance assigned to each skill and perceived self-performance scores.

**Table 4:** **Junior researchers’ perception of barriers in subjective sub competencies for five disciplines**

***(n = 46)***	***Importance score mean ± SD***	***Performance score mean ± SD***	***Gap (difference) mean ± SD***	***Training need %***	***Familiarity %***
***Health economics***	***5.8 ± 1.3***	***2.4 ± 1.3***	***3.4 ± 1.5***	***78***	***57***
Statistical and econometric analysis	5.8 ± 1.3	2.0 ± 1.3	3.8 ± 1.7	84	48
Microeconomics of health care	5.7 ± 1.5	2.0 ± 1.3	3.6 ± 1.8	82	41
Health accounting	5.8 ± 1.5	2.3 ± 1.2	3.5 ± 1.7	75	55
Economic evaluation	5.9 ± 1.4	2.4 ± 1.5	3.4 ± 1.9	75	59
Health Financing functions	5.9 ± 1.3	2.6 ± 1.5	3.2 ± 1.6	75	66
Economics of health systems	6.1 ± 1.3	2.9 ± 1.7	3.2 ± 1.7	77	73
Provider payment mechanisms	5.7 ± 1.6	2.6 ± 1.6	3.0 ± 1.7	77	57
***Health policy***	***6.0 ± 1.3***	***2.9 ± 1.5***	***3.1 ± 1.6***	***84***	***72***
Monitoring and evaluation methods	5.9 ± 1.5	2.6 ± 1.6	3.3 ± 1.8	84	57
Health policy analysis frameworks	5.8 ± 1.5	2.5 ± 1.6	3.3 ± 1.9	84	64
Policy processes in health care	6.2 ± 1.3	3.1 ± 1.6	3.1 ± 1.7	82	77
Impact of policies on population	6.1 ± 1.3	3.0 ± 1.6	3.0 ± 1.7	84	82
Political influence on resource alloc.	5.9 ± 1.4	3.0 ± 1.6	3.0 ± 1.8	82	71
Organization, financing & health syst.	6.2 ± 1.3	3.3 ± 1.7	2.9 ± 1.7	86	82
***Environmental health***	***6.2 ± 0.9***	***3.2 ± 1.6***	***3.0 ± 1.6***	***76***	***74***
Environmental epidemiology	6.2 ± 1.0	2.9 ± 1.7	3.4 ± 1.7	82	64
Policies to mitigate env. hazards	6.3 ± 1.1	3.0 ± 1.7	3.3 ± 1.8	75	73
Interaction of environ. determinants	6.1 ± 1.1	2.9 ± 1.5	3.2 ± 1.8	80	75
Health & environ. risk assessment	6.3 ± 1.0	3.2 ± 1.7	3.1 ± 1.7	80	75
Factors modifying impact of env.	6.1 ± 1.1	3.1 ± 1.8	3.0 ± 1.8	75	71
Exposure assessment methods	6.1 ± 1.1	3.2 ± 1.9	2.9 ± 1.8	77	71
Sources, pathways, of exposure	6.3 ± 0.9	3.5 ± 1.9	2.8 ± 1.7	71	80
Major environ. & occup. hazards	6.4 ± 1.0	3.6 ± 1.9	2.7 ± 1.8	68	82
***Medical anthrop & sociology***	***5.9 ± 1.2***	***3.0 ± 1.5***	***2.9 ± 1.6***	***75***	***63***
Ethnographic methods	5.6 ± 1.5	2.4 ± 1.7	3.2 ± 1.8	77	34
Health seeking behavior	6.2 ± 1.1	3.2 ± 1.8	3.0 ± 1.8	77	71
Historical & political dimensions	5.9 ± 1.2	2.9 ± 1.8	3.0 ± 1.8	71	59
The clinic/hospital as social	5.6 ± 1.6	2.7 ± 1.6	2.9 ± 1.7	75	59
Role of culture in health	5.9 ± 1.3	3.1 ± 1.8	2.8 ± 1.9	77	66
Social inequalities in health	6.3 ± 1.1	3.7 ± 1.8	2.6 ± 1.9	73	84
Understanding popular health	5.8 ± 1.5	3.2 ± 1.7	2.6 ± 1.8	73	71
***Epidemiology***	***6.3 ± 0.7***	***4.3 ± 1.4***	***2.0 ± 1.6***	***73***	***87***
Mathematical modeling	6.0 ± 1.2	3.0 ± 1.5	3.0 ± 1.9	89	66
Disease surveillance	6.4 ± 0.8	4.0 ± 1.5	2.4 ± 1.6	82	86
Methods in epidemiology	6.6 ± 0.7	4.5 ± 1.8	2.1 ± 1.7	64	93
Subjective health measures	5.9 ± 1.1	3.8 ± 1.7	2.1 ± 1.7	73	84
Effect modification (confounding)	6.2 ± 1.0	4.2 ± 1.7	2.0 ± 1.9	77	82
Risk factors and susceptibility	6.5 ± 0.8	4.5 ± 1.7	2.0 ± 1.8	77	89
Validity and reliability	6.4 ± 0.8	4.4 ± 1.7	2.0 ± 1.9	71	89
Association and causation	6.4 ± 0.8	4.6 ± 1.8	1.9 ± 1.8	68	91
Statistical analysis of data	6.5 ± 0.8	4.7 ± 1.7	1.8 ± 1.8	71	96
Measures of morbidity & mortality	6.3 ± 1.0	4.8 ± 1.8	1.4 ± 1.8	61	96

Table 4 indicates the discipline-specific skills and the component skills associated with each of the five disciplines and their identified sub-competencies, which are the selected focus of the larger research project. Five disciplines are Health Economics, Health Policy, Environmental Health, Medical Anthropology & Sociology, and Epidemiology. Each discipline has six to ten sub-competencies that junior researchers rated regarding importance, level of knowledge appearing as performance score, how familiar they are with the area, how much training they need, and a score we calculated as the gap between importance and performance scores.

The averages of performance scores of junior researchers for five specific disciplines were usually below 4 points, except for epidemiology. This means that junior researchers need epidemiology training the least, except in three sub competencies; mathematical modeling, disease surveillance and subjective health measures, all of which were below 4 points (Table 4). The biggest gap among the main disciplines was observed in relation to health economics (3.4 points), followed by health policy, environmental health and medical anthropology/sociology (ranking as 3.1, 3.0, and 2.9 points respectively).

The biggest gap for all sub competencies was in “statistical and econometric analyses” (3.8), followed by “micro economics of health care” (3.6) and “health accounting” (3.5), all housed under Health Economics. When asked about their training needs, 89% of junior researchers indicated a need for training in “mathematical modeling” (under Epidemiology) and 86% in “organization, financing and delivery of health services and public health systems” (under Health Policy). The lowest training need identified by participants are “measures of morbidity and mortality” with 61%, “methods in epidemiology” with 64%, “association and causation” with 68% (all under Epidemiology), and “major environmental and occupational hazards” with 68% (under Environmental Health).

The final column in Table 4, labeled “familiarity” shows that junior researchers in our study are highly familiar with Epidemiology (87%) and Environmental Health (74%) and least familiar with Health Economics in disciplinary categorization, however the least familiar sub-competencies are not from Health Economics. Among sub-competencies, researchers are least familiar with “ethnographic methods” (with 34%, under Medical Anthropology & Sociology), followed by “micro economics of health care” (with 41% under Health Economics).

**Figure 2:**
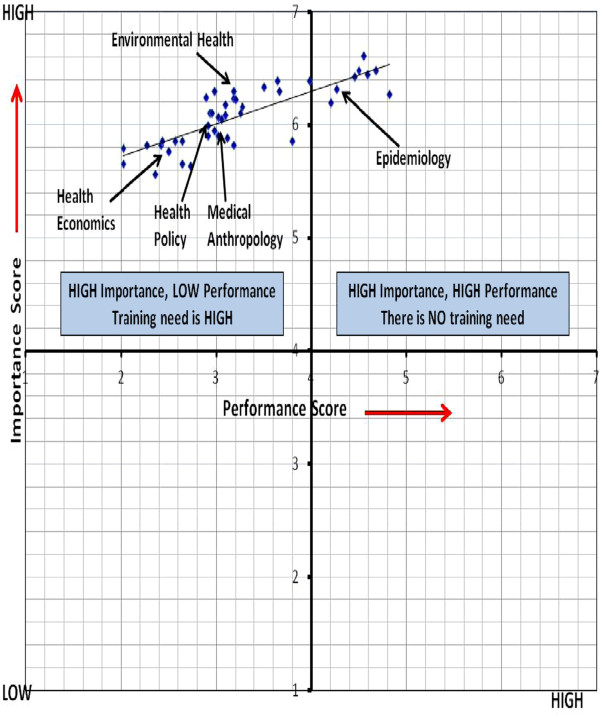
**Comparing importance and performance scores for the 5 disciplines and 38 sub competencies.**

One may expect to see the highest training need in areas researchers are the least familiar with, however that is not necessarily the case. For example, training need stated for “ethnographic methods” and “health seeking behavior” were both 77%, however familiarity was lowest in “ethnographic methods” (with 34%), while it was 71% for “health seeking behavior.” In general, junior researchers in our study stated a need for training in all disciplinary areas, ranging between 73% and 84%, which are high and partly may explain the cases above. It makes more sense to look at the importance scores in conjunction with performance scores to understand training need rather than using familiarity. Hence looking at these three columns together provides more information in explaining the differences between similar training need assigned to different familiarity scores or vice versa (Table 4). To understand these distinctions more clearly, we offer the following plot graph in Figure 2.

Training needs for the 5 disciplines and 38 sub-competencies are plotted according to their importance and performance scores as illustrated in Figure 2. This quadrant graph form makes the training needs in the various competencies clear. Comparing scores for importance and performance indicates where the greatest training needs lie, with the biggest gap indicating the greatest training needs. Any sub competency item may be given a score of 4 or above for importance, less than 4 for performance. This would indicate that this is an urgent training need (upper left quadrant). An item with importance and performance scores of less than 4 would suggest a training need, but not an urgent one (lower left quadrant). Of course, an item with a performance score of four or above indicates that performance on this item is satisfactory and no intervention required [22].

Health economics, health policy, environmental health and medical anthropology disciplines and their sub competencies were given a score of over 4 for importance and below 4 for performance. This means that there is an urgent training need (left upper quadrant) for these disciplines. Average performance scores of junior researchers for epidemiology were over 4 points, except for mathematical modelling, disease surveillance and subjective health measures. This means that junior researchers do not need epidemiology training except in these three sub competencies.

The Training Needs Assessment (TNA) survey also included questions about barriers against research. According to junior researchers, the reasons for not being able to conduct research in the five main disciplines were firstly, not having enough knowledge (68%), secondly, this not being a priority area for the institution (53%) and thirdly, not having enough time (32%). Eighty-two percent of junior researchers mentioned that they had to provide health services while at same time they did research, and 31% of them were also teaching. This is in agreement with what the KIs in interviews mentioned about heavy workloads and doing too many things as part of the workday rather than specialization and prioritization.

Regarding training, participants’ most preferred method was courses with certification (83%). Other methods preferred were short-courses completed over a short period of time (70%), e-learning (61%), mentorship (61%) and short-term courses completed over a long period of time (50%).

According to 44% of the sample, it was inconvenient to attend courses abroad, particularly for MoH staff due to securing work release.

Additionally, TNA inquired about participants’ disciplinary priority areas. This question was an open ended one, which required participants to write in top priority disciplines. We calculated percentages for their top choice, which is presented in the table below. First priority areas identified by junior researchers are epidemiology (34.7%), health policy (19.5%), health economics (11%), medical anthropology & sociology (6%) and environmental health (6%) (Table 5). Though the highest and lowest priority disciplines identified by KIs and junior researchers are the same (epidemiology and environmental health respectively), the middle rankings do not match. KIs identified the disciplines and

**Table 5:** **Disciplines related to training areas, according to KIs, and junior researchers**

Rank	Training areas and sources			
	Key informants (n:10)	Junior researchers (n:46)		
	(n/total number)	First priority discipline (% )	Willing to conduct research (%)	The gap* (mean)
**1.**	Epidemiology (7/10)	Epidemiology (35%)	Epidemiology (85%)	Health economics (3.4)
**2.**	Medical anthropology and sociology (7/10)	Health policy (19%)	Health policy (72%)	Health policy (3.1)
**3.**	Health policy (5/10)	Health economics (11%)	Medical anthropology and sociology (72%)	Environmental health (3.0)
**4.**	Health economics (5/10)	Medical anthropology and sociology (6%)	Health economics (67%)	Medical anthropology and sociology (2.9)
**5.**	Environmental health (3/10)	Environmental health (6%)	Environmental health (54%)	Epidemiology (2.1)

*The gap represents the difference between the importance and performance scores.

sub-competencies with a training need as advanced epidemiology (surveillance, burden of disease, modelling, community based interventions, with 70%), medical anthropology/sociology (qualitative research techniques, behavior change, social determinants of health, with 70%), health policy (policy processes, health systems, planning, with 50%), health economics (cost effectiveness, cost determining, with 50%) and environmental health (with 30%). While KIs suggested training in medical anthropology and sociology as one of the highest disciplinary need, junior researchers ranked this need as one of the last priorities (6%). This contrast between KIs and junior researchers suggest that KIs are able to provide an overview of the disciplines and the research base, and are able to pinpoint the lack of attention to a much needed social science approach in health research. KIs’ focus can be useful in explaining the value of ethnographic methods for identifying popular health concepts and health seeking behaviour to junior researchers. Junior researchers, on the other hand, focus on their immediate needs, as they are able to evaluate them with greater precision, and also prioritize what they need the most (Table 5).

The last area of inquiry in the survey was the willingness to pursue research by discipline. This question was also an open ended one, which asked participants to rank disciplines. Eighty-five percent of junior researchers are willing to conduct research in epidemiology, which is the highest ranked research discipline in terms of willingness. This finding agrees with regarding priority disciplines to receive further training. Junior researchers were also willing to conduct research in health policy (72%), medical anthropology (72%) and health economics (67%). Junior researchers were least willing to conduct research in environmental health (54%). According to the participants, their institutional attitudes are largely positive towards epidemiology (72%). However, the percentage of perceived institutional willingness reported was below 50% for all other disciplines according to KIs.

## Discussion

In order to suggest improvements to national health research capacity in the field of NCDs, it is necessary to evaluate the current situation in Turkey. In the first stage of this study, we obtained a list of papers on NCDs from PubMed; 632 articles published by 692 researchers in the past 12 years. This means that only 1.1 article per 10 researchers has been published over the last 12 years (53 articles per year). This is a small number for Turkey when we compare it to countries of similar size. Even more starkly, only 5% of all papers (31 in total) have any kind of focus on inequalities and the social determinants of health, an area of public health research which has been largely neglected in Turkey up till now. This points to a lack of emphasis placed by researchers on the field of health inequalities and social determinants of NCDs. The majority of papers were about hypertension and diabetes mellitus, while there were only 13 papers on the subject of physical activity. It seems that research on physical activity is very weak in Turkey and should be improved as well. On the other hand, when we examine the total number of scientific journal articles in one year, we see 8,301 articles in 2009 in Turkey. The total number of scientific journal articles in one year was over 45,000 in the UK and Germany, 31,748 in France, and 21,543 in Spain [6]. This comparison reveals a general lack of research productivity in Turkey.

According to our findings, both the KIs and junior researchers assessed research capacity building and training in Turkey as very important. However, the judgements of KIs and junior researchers were occasionally contradictory. KIs seem to be looking at the wider context in which capacity building occurs. For example; KIs see the barriers as relating to a lack of structures in which to make use of these capacities once they have been acquired while junior researchers mention their training needs. KIs refer to the disincentives for research: because the emphasis in universities is on teaching, this leaves little time for research. Teaching suits generalists rather than specialists, thereby reducing incentives for the kind of specialization research requires. However some organizational/managerial and psychological/motivational problems like job satisfaction/job stress may be additional barriers which contribute to differences between the perceptions of KIs and junior researchers. There is also a deficit in the relationship between the MoH and academia, with ill-defined research priorities. In addition, English being the language of research itself creates a barrier to capacity-building. The question of morale also comes into this. Finally, there is the issue of funding, and we have two very insightful quotes about the financial disincentives for research: the negative effects of new performance based payment system and the decline in support from pharmaceutical companies. Additionally, when we compare with the other project countries, Turkey reported highest mean level of training needs in all disciplines. This could be due to higher importance scores given in Turkey compared with other countries [21]. Our findings are important in determining the priority areas for training junior researchers.

Other challenges for capacity building in Turkey mentioned by the KIs were lack of coordination between institutions and researchers, lack of monitoring and evaluation systems for research, and lack of routine health information systems which can provide data for operational research. However, KIs indicated that the main reason for these challenges was a lack of health research priority areas at national level, and utilization, dissemination and promotion of research in NCDs. This is connected to the need for national and international networks. Review of low and middle income country cooperation strategies showed that NCD research policies (prioritization of implementation research, strengthening research capacity and resource allocation) in the national NCD agenda are very weak. Only 32% of low and middle income countries (n:61) refer to policies to facilitate NCD researches [31].

Epidemiologists working in state and territorial departments in the USA reported that they needed additional training in the following main areas: evaluation of public health interventions (93%), designing epidemiological studies (83%), leadership and management training (80%), analyzing epidemiological data with statistical software (80%), surveillance systems (79%), and designing data collection tools to address a health problem (79%) [32]. These are very similar to our findings in Turkey. Since 1991, there has been an increase in epidemiological research productivity, which is the result of the implementation of a number of epidemiology programs in WHO/AFRO region [33]. However this increase does not compare to the Western countries’ productivity. The application of epidemiology “to control health problems” appears to be of primary interest. In this sense, “epidemiological research” is often used synonymously with “public health research”. To further increase research productivity, an increase in epidemiology education and training programs are needed [33]. Increased need for epidemiology and epidemiologists is true for developed nations as well. For example, in 2004, in state and territorial health departments in USA; a survey estimated that the number of employed epidemiologists should be increased by 47% to address the problem of chronic disease, and by 51% among environmental health experts [32]. Boulton also suggests that the state health departments need 68% more epidemiologists to reach optimal capacity in all program areas [34]. Our study similarly found that epidemiology ranks as the most prioritized discipline among all of our participants.

Public health research can feed into health policy and therefore an increased public health research capacity is part of this evolution. Research on health systems and policy relevant research in ten countries in the Eastern Mediterranean Region indicate the importance of disseminating results to other researchers, and to policymakers [35]. Insufficient policy dialogue opportunities and collaboration between researchers and policymakers and stakeholders hinder the use of evidence. The most frequently mentioned barrier to use of evidence in policymaking is lack of funding for research, and the most frequently mentioned enabler is communication and networking [35]. Health policymakers from ten countries in this project recognize the importance of using health systems evidence in health policymaking. Most of them report requesting evidence and nearly half of them report that research evidence is not delivered at the right time, and that there is lack of collaboration with researchers, a lack of explicit budget, and a lack of administrative structure – all of which limits the use of research evidence [36]. The Turkish health research system similarly lacks intersectoral cooperation. A national health research system has a wide range of actors and institutions from the public and private sectors, NGOs and academia. There are five specific stakeholder groups in this system: policymakers and managers, health professionals, patients, industry and researchers. Researcher needs include resources for research, its dissemination, control and independence in the research process [9].

Health policy and system priority research topics were identified via a survey conducted between 2000 and 2002 across developing countries. The highest ranking topic was “sector analysis” followed by “disease burden”, “management and organization”, “program evaluation”, “accessibility”, “research to evidence” and “financing” [37], which is similar to our findings. In our study, the training areas within health policy are identified as monitoring and evaluation methods, frameworks of health policy analysis, policy processes and impact of policies on population. Regarding this interplay between policy making and research, the literature suggests that engaging policymakers and stakeholders in research priority-setting exercises increase the likelihood of the utilization of research evidence by policymakers [5, 38]. Health research priority setting processes assist researchers and policymakers in effectively targeting research that has the greatest potential public health benefit. Many different approaches to health research prioritization exist (e.g. the checklist suggested by Viergaver [39]), but there is no agreement on what might constitute best practice. According to Council on Health Research for Development (COHRED), research priority areas should be research capacity strengthening, epidemiology of most common diseases, health care financing, health systems and policy analysis, effects of environmental and social factors, and cost-benefit analyses of health policies [38]. These are similar to our findings. Additionally, the competencies required of public health workers were similarly defined in the early 1990s in USA, where the public health faculty/agency forum recognized six disciplines as primary: analysis (biostatistics), basic science (epidemiology, NCDs, etc), finance and management, policy and program planning, culture and communications [40]. These results resonate with the five main disciplines in our study as well as with what our participants mentioned as significant.

We recommend specific training for junior researchers in the topics of advanced epidemiology, health policy, health economics, medical sociology, medical anthropology, environmental health and English language competency. Networking between researchers should be facilitated. We recommend an immediate revision of the reorganization of the General Directorate of Health Research in the MoH to ensure that the staff works in appropriate positions commensurate with their training where they can apply their knowledge immediately following the training. Specialization should be encouraged and therefore heavy teaching loads should be lightened in academic institutions. English training should be prioritized for junior researchers. Performance based payments should include research activities.

### Limitations

This research has three main limitations. First, this is a small study, undertaken as the prelude to a larger programme of research capacity building (RESCAP-MED). Second, the selection of junior researchers was based on the key informants’ suggestions, which creates its own bias. We cannot therefore claim that this sample is representative of all junior researchers in Turkey. It is however indicative. Third, qualitative research studies in the field of medicine are still uncommon in Turkey, and there are few examples to build upon. Despite these limitations, this study is innovative, and has provided data and insights on which future research in Turkey can build.

## Conclusion

In conclusion, there is great need for training of junior researchers in Turkey. However training by itself is not sufficient. Lack of coordination between governmental institutions and researchers is the main problem facing capacity building among junior researchers. Research monitoring and evaluation systems are not sufficient, while routine health information systems are also inadequate. Dissemination and promotion of research results is weak. Specialization is not common in departments of public health. Financial resources for research activities are not sufficient. To create an impetus for research productivity, a priority research topics list can be determined at the national level by the Turkish MoH, and the General Directorate of Health Research. In conjunction with an increased budget, specifically for research, this list can be instrumental in communicating to researchers which subject areas would receive priority for distribution of financial resources. Such prioritization can also encourage the closing of the gap in areas, which do not receive research attention, such as the social determinants of health.

Although this was a small-scale project, designed to establish baseline data for a wider research capacity-building project, it is nevertheless the most extensive exercise, undertaken either in Turkey or the wider region, in assessing research capacity and training needs relevant to the growing burden of NCDs. There are considerable strengths to build upon in Turkey. However a combination of institutional disincentives for research, and the lack of opportunities for the rising generation of researchers to acquire advanced training skills, still hamper development of a research base appropriate to Turkey’s size and aspirations.

Though our study is specific to the Turkish case, implications for the wider context are apparent. NCDs are a concern in all countries including developed nations. How to establish training protocols, establishing baseline data for policy suggestions and future program evaluations are important concerns in this area of research. The Turkish case is particularly relevant to developing countries and the southern Mediterranean and Middle Eastern regions. Concerns identified in this study, such as importance of applied research, policy implications and collaboration among stakeholders, are applicable to a wider range of contexts beyond Turkey. Lack of vision for and financial resources devoted to research capacity building in public health plague many national settings, not just Turkey. It is our hope that, concerns raised by our participants regarding establishing research and training in public health disciplines, also echoed by the Committee on Health Research [2], will receive the structural attention it deserves.

## Abbreviations

COHRED: Council on Health Research for Development
DALY: Disability Adjusted Life Years
DM: Diabetes Mellitus
KI: Key Informant
MedCHAMPS: Mediterranean studies of Cardiovascular disease and Hyperglycaemia: analytical Modelling of Population Socio-economic transitions
MoH: Ministry of Health
NCD: Non communicable diseases
NGO: Non-governmental organizations
RESCAP-Med: NCDs and their social determinants in Mediterranean partner countries: building sustainable research capacity for effective policy intervention
TNA: Training Needs Assessment
TUBITAK: Scientific and Technological Research Council of Turkey
TUIK: The Turkish Statistical Institution
UNDP: The United Nations Development Program
WHO: The World Health Organization
YÖK: Higher Education Council of Turkey.
